# The Focal Adhesion: A Regulated Component of Aortic Stiffness

**DOI:** 10.1371/journal.pone.0062461

**Published:** 2013-04-23

**Authors:** Robert J. Saphirstein, Yuan Z. Gao, Mikkel H. Jensen, Cynthia M. Gallant, Susanne Vetterkind, Jeffrey R. Moore, Kathleen G. Morgan

**Affiliations:** 1 Department of Biomedical Engineering, Boston University, Boston, Massachusetts, United States of America; 2 Department of Health Sciences, Boston University, Boston, Massachusetts, United States of America; 3 Department of Physics, Boston University, Boston, Massachusetts, United States of America; 4 Department of Physiology and Biophysics, Boston University Medical School, Boston, Massachusetts, United States of America; University of California, San Diego, United States of America

## Abstract

Increased aortic stiffness is an acknowledged predictor and cause of cardiovascular disease. The sources and mechanisms of vascular stiffness are not well understood, although the extracellular matrix (ECM) has been assumed to be a major component. We tested here the hypothesis that the focal adhesions (FAs) connecting the cortical cytoskeleton of vascular smooth muscle cells (VSMCs) to the matrix in the aortic wall are a component of aortic stiffness and that this component is dynamically regulated. First, we examined a model system in which magnetic tweezers could be used to monitor cellular cortical stiffness, serum-starved A7r5 aortic smooth muscle cells. Lysophosphatidic acid (LPA), an activator of myosin that increases cell contractility, increased cortical stiffness. A small molecule inhibitor of Src-dependent FA recycling, PP2, was found to significantly inhibit LPA-induced increases in cortical stiffness, as well as tension-induced increases in FA size. To directly test the applicability of these results to force and stiffness development at the level of vascular tissue, we monitored mouse aorta ring stiffness with small sinusoidal length oscillations during agonist-induced contraction. The alpha-agonist phenylephrine, which also increases myosin activation and contractility, increased tissue stress and stiffness in a PP2- and FAK inhibitor 14-attenuated manner. Subsequent phosphotyrosine screening and follow-up with phosphosite-specific antibodies confirmed that the effects of PP2 and FAK inhibitor 14 in vascular tissue involve FA proteins, including FAK, CAS, and paxillin. Thus, in the present study we identify, for the first time, the FA of the VSMC, in particular the FAK-Src signaling complex, as a significant subcellular regulator of aortic stiffness and stress.

## Introduction

Normal cardiovascular function depends on the biomechanical properties of blood vessels, and changes in these properties are characteristic of disease. Increased aortic stiffness precedes and predicts for the development of hypertension and subsequent negative cardiovascular outcomes, including myocardial infarction, stroke, and heart disease [1], [2]. A stiffer aorta deforms less in response to pulse pressure, absorbing less of the pulse while transmitting a greater portion of the potentially harmful pulsatile energy downstream to delicate vessels of the microcirculation in the heart, lung, brain and kidneys [1].

The mechanical properties of a blood vessel, like any material or tissue, are determined by its components and their organization. Accordingly, the stiffness of vascular tissue can be derived from the composition of the extracellular matrix (ECM) and the cells in the tissue, the organization of the matrix and cells, and the connections that link these components (cell-matrix and cell-cell contacts). The prevailing view is that the ECM is the principal determinant of vascular stiffness [3]; however, the extent to which other tissue components contribute is unclear.

Vascular smooth muscle cells (VSMCs), the predominant cell type in the wall of large blood vessels, influence tissue stiffness when modulating contractile tone or when depositing matrix or matrix-degrading enzymes [3], [4]. Recently, the stiffness of VSMCs themselves has been implicated as an important determinant of tissue stiffness [5], with the actin cytoskeleton suggested as the specific cellular component responsible. Based on this finding, as well as the recent finding that the cortical actin cytoskeleton is dynamic in vascular smooth muscle tissue [6], we hypothesized that the cortical focal adhesions (FAs) (also known as adhesion plaques or dense plaques in smooth muscle tissues [7]) may be an important, and possibly regulated, component of vascular tissue stiffness. Here we define the FA to include its attachments to the cortical actin cytoskeleton as well as the integrins that attach the cell to the ECM.

In the present study, we examine the mechanical properties of blood vessels across multiple length scales and identify, for the first time, the Src-FAK signaling complex of FAs of the non-migratory, non-proliferating VSMCs embedded in the blood vessel wall as a significant regulator of aortic stiffness. Finally, we show that FA size, cortical stiffness, and VSMC contractility influence the FA-regulated component of aortic stiffness.

## Methods

### Ethical Approval

All procedures were performed in accordance with protocols approved by the Boston University Institutional Animal Care and Use Committee (Permit Number: A3316-01). The animals were maintained according to guidelines in the NIH Guide for the Care and Use of Laboratory Animals and were obtained and used in compliance with federal, state, and local laws. Tissue removal was quickly performed after isoflurane euthanasia.

### Cell Culture

A7r5 vascular smooth muscle cells, originally derived from rat aorta (American Type Culture Collection, Manassas, VA), were maintained as described previously [8]. A7r5 cells proliferate as myoblasts and, when they reach the stationary phase, upon serum deprivation, differentiate into a phenotype that resembles adult smooth muscle cells [9], [10]. The cells express many key markers of smooth muscle, including smooth muscle α-actin, smooth muscle myosin, smooth muscle tropomyosin isoforms, h1 calponin, and SM22 α [11]. This approach was chosen over primary cultured cells because of the variability of differentiation state with passages. For all experiments, A7r5 cells were seeded onto coverslips or, for magnetic tweezers experiments, 35-mm glass-bottom culture dishes (MatTek, Ashland, MA), grown to 75–90% confluence in the presence of serum and then serum-starved for 24 hours to trigger differentiation to the quiescent (non-proliferating, non-migrating) smooth muscle phenotype.

### Bead Coating and Attachment

For magnetic tweezer studies, an arginine-glycine-aspartic acid (RGD) peptide (GRGDNP, Enzo Life Sciences, Farmingdale, NY) was covalently coupled to amine-modified superparamagnetic Dynabeads M-270 (2.8 µm, Invitrogen, Grand Island, NY) according to a protocol from Bangs Laboratories (TechNote 205: Covalent Coupling, Bangs Laboratories, Inc., Fishers, IN). Briefly, amine-modified Dynabeads were suspended in phosphate buffered saline (10 mg/ml), activated with 10% glutaraldehyde, reacted with RGD peptide (0.5 mg/ml), quenched with 40 mM glycine and blocking agent, and stored with blocking agent and preservative in phosphate buffered saline.

RGD is an ECM protein motif that is a minimal sequence required for binding by transmembrane integrin receptors [12]. The GRGDNP peptide has affinity for the α_5_β_1_ and α_v_β_3_ integrins, which are present in vascular smooth muscle tissues and recognize fibronectin and vitronectin, respectively [12]–[14].

Before making measurements with the magnetic tweezers, the beads were washed and sonicated, and then 6.7×10^5^ beads were added to the 35-mm glass bottom dish containing cells. The beads were incubated with the cells for 30 min at 37**°**C with 5% CO_2_ to facilitate their attachment to the cells. Following this incubation, the serum-free medium was exchanged twice to remove unbound beads. Measurements of cortical stiffness were performed at room temperature. Measuring time was limited to 30 minutes per dish.

### Measurement of Stiffness with Magnetic Tweezers

The magnetic tweezers (MT) apparatus is modeled after a system designed by the Fabry group [15], and it consists of a magnetic microneedle made from a cylindrical rod of high-permeability, low-hysteresivity nickel alloy (HyMU-80, Carpenter Technology, Reading, PA) with a 4.5-mm core diameter and its needle-end sharpened to a 40-µm radius (courtesy of the Boston University machine shop); a solenoid (200 turns of 0.5 mm copper wire) surrounding the microneedle; and a power supply (MPJA, Lake Park, FL) connected to the solenoid.

Calibration of the magnetic tweezers was performed similarly to what has been done by others [15]. Briefly, the movements of beads through a viscous mixture of 3∶1 glycerol to ddH_2_O (viscosity *η* = 0.052 Pa s at room temperature) were used to compute forces on the beads for different solenoid currents using Stokes’ formula for viscous drag, *F* = 6π*ηrv*, where *η* is the viscosity, *r* is the radius of the bead, and *v* is the velocity of the bead through the mixture. The calibration data for all beads were then fit to a simple empirical equation relating the force on a bead to both the bead’s distance from the magnetic tweezers tip and the current through the solenoid [15]. This relationship was then used to analyze data from cortical stiffness experiments and calculate forces exerted on cell-bound beads based on their distance from the MT tip and the set current.

To measure cortical stiffness, the MT tip is positioned approximately 150–200 µm from a cell-adherent bead using a micromanipulator. This distance was chosen because the force-distance relationship is highly nonlinear at short bead-tip distances but flattens out at distances greater than 100 µm. Thus, at an operating distance of 150–200 µm, a pull on a bead will displace it towards the tip and only negligibly increase the force exerted by the MT on the bead (i.e., force is essentially constant, F ∼ 50 pN). An operating current of 1.5 A was chosen for experiments because it produces low forces appropriate for cortical probing [16]–[19]. At this low current (and for the short pull durations employed), there is no significant heating of the rod or the sample. Between measurements, the stage is displaced a distance of at least 0.5 mm so that cells do not experience forces from previous measurements. Beads selected for analysis are less than 50 µm from the cell edge and bound on the MT-side of the cell, sufficiently far from the nucleus, to avoid pulling the bead uphill. Additionally, beads were selected for pulling only if there were no overlapping cells in the vicinity of the bead that could influence the local apical topography of the cell.

Cortical stiffness, which we define as the stiffness of the bead-integrin attachment and the underlying FA and cortical cytoskeleton, was measured by employing a priming protocol described by others that consists of a series of five identical square-wave cycles that are 4 sec in duration and 4 sec apart [17], [20]. Each pull displaces the bead towards the MT probe, and in between pulls the bead recoils and reaches equilibrium. The bead does not return to its exact start position with each pull, leading to a shift in the baseline with each pulse that stabilizes within about three pulls. The fifth and final pull is analyzed to measure cortical stiffness (calculated as a ratio of the force applied to the displacement of the cell-adherent bead).

Experiments were performed at 20× magnification (NA 0.5) with a Nikon Plan Fluor objective under bright-field illumination on an Eclipse TE2000-E inverted microscope (Nikon Instruments). Videos of beads and the MT tip were recorded in real time with a charge-coupled device camera (CoolSNAP HQ^2^, Photometrics) at 10 frames per second using the Nikon NIS Elements imaging software (Nikon Instruments). Positions of beads were tracked with an intensity-weighted center-of-mass algorithm using the MTrackJ plug-in for ImageJ [21] and later analyzed in Matlab (Natick, MA).

Lysophosphatidic acid (LPA, 10 µM) was added for 15 minutes, PP2 (10 µM) was added for 30 minutes, or PP2 was added for 30 minutes followed by LPA for 15 minutes. Experiments were performed at room temperature, and measurement time with the MT for each sample was limited to 30 minutes to ensure cell viability.

### Immunofluorescence Imaging and Analysis

A7r5 cells were fixed and stained as previously described [8]. Nuclei were stained with 4,6-diamidino-2-phenylindole (Sigma-Aldrich, St. Louis, MO), and filamentous actin was stained with Alexa Fluor 568 and 488 phalloidin (1∶3000, Invitrogen). Cells were examined with an Eclipse TE2000-E fluorescence microscope (Nikon Instruments) equipped with a Nikon Plan Apochromat 60X (NA 1.4) oil immersion objective, a charge-coupled device camera (CoolSNAP HQ^2^, Photometrics), and filters optimized for double-label experiments. NIS-Element Advanced Research software (Nikon Instruments) was used to capture images and for removal of out-of-focus fluorescent blur by deconvolution of Z-stacks (Richardson-Lucy algorithm, constrained iterative-maximum likelihood estimation algorithm) as previously described [22]. Subsequent processing was completed with Photoshop CS3 software (Adobe Systems, Mountain View, CA).

Areas of individual FAs were determined using NIS-Elements Advanced Research software. FAs were measured for 5 to 13 A7r5 cells per condition (control, PP2, LPA, PP2+ LPA) per experiment (total n = 82 cells). In each cell that was selected for analysis, all peripheral FAs were examined for analysis because these adhesions are most relevant to the question of force transmission, as numerous studies have reported that the largest traction forces are found in the cell periphery [23]. Cells with overlapping neighbors that would obscure measurements were excluded from the analysis.

### Aorta Tissue Preparation

C57bl/6 Mice (*Mus musculus*) (3 months old, n = 31) (Charles River, Wilmington, MA) were euthanized with an overdose of isoflurane by inhalation. The aorta was promptly excised, rinsed six times to remove blood within or around the vessel, which can harm the smooth muscle cells and induce vasoconstriction [24], and stored in an oxygenated (95% O_2_–5% CO_2_) physiological salt solution (PSS) (in mM: 120 NaCl, 5.9 KCl, 1.2 NaH_2_PO_4_, 25 NaHCO_3_, 11.5 dextrose, 1CaCl_2_, and 1.4 MgCl_2_; pH = 7.4). Following careful dissection in oxygenated PSS to remove connective tissue and adventitia, aortic rings were cut (4 mm long) then suspended *in vitro* in tissue baths containing oxygenated PSS at 37°C. Smaller rings immediately proximal and distal to these suspended segments were also cut for determining ring thickness. Following the measurements made in the tissue baths, aortic rings were quick-frozen at −78.5°C in a slurry of dry ice and liquid acetone containing 10 mM dithiothreitol and 10% TCA for subsequent biochemical analysis [25]. The TCA functions to denature and deactivate endogenous kinases, phosphatases, and proteolytic enzymes, thereby preserving the levels of phosphorylation present at the time of freezing [26], [27]. Freezing of the thin-walled mouse aorta rings occurs rapidly in comparison to the minute timescale for phosphorylation events, contraction, or relaxation.

### Measurement of Tissue Thickness

Tissue thickness was measured to determine cross-sectional area for stiffness calculations. Thin tissue rings were cut from both ends of an aorta ring segment prior to measuring its stiffness in the tissue bath. The thin rings were incubated with 2 drops/ml of NucBlue (Life Technologies, Grand Island, NY), a vital nuclear stain, for 30 minutes. Each ring was placed in a small PSS-filled chamber on a slide with the major axis of the vessel perpendicular to the slide for examination in cross-section. Images were taken under the blue channel (cell nuclei) and the green channel (autofluorescent internal elastic laminae) at 20x, moving around the ring to acquire a total of six images for thickness measurements. For each of the six images, four line segments were arbitrarily drawn to measure stiffness at different positions on the vessel wall. All twenty-four measurements per ring were averaged. Then, the thicknesses of the two bordering thin rings were averaged to determine the mean thickness of the aorta ring segment (∼60 µm).

### Tissue Stiffness Measurements with Sinusoidal Perturbations

For *in vitro* force and stiffness measurements, thin triangular shaped wire clasps (d = 0.005 in) were threaded through the lumen of the aorta rings then attached at one end to a fixed hook and at the other end to a computer-controlled motorized lever arm (Dual-Mode Lever Arm System, Model 300C, Aurora Scientific, Ontario, Canada) capable of setting tissue length while simultaneously measuring force. The rings were stretched uniaxially in the circumferential direction, as vascular smooth muscle cells in the aorta wall are oriented primarily in this direction, and this is the primary direction of strain induced by pulsatile flow *in vivo*.

Rings were stretched to optimal length *L*_O_ (1.8 x slack length, or equivalently a strain ε = 80%) for 30 minutes to allow adequate time for stress relaxation (i.e., for tensile force to stabilize at a steady state level). The optimal length for maximal contraction was predetermined by length-tension curves (data not shown); for mouse aorta, *L_O_* is near the upper end of the tissue’s physiologic strain range during systolic/diastolic pressure transients [28]. Following this step, the tissue was stimulated to contract for 15 minutes by depolarization with physiologic saline solution in which 51 mM NaCl had been replaced by KCl, followed by a series of three washouts with regular PSS and a 30 minute relaxation period. The observation of the magnitude of the KCl contractions confirms viability and adequate equilibration of the tissue. Then rings were incubated with vehicle (DMSO, 1∶1,000; or ethanol 1∶1,000), PP2 (10 µM), FAK inhibitor-14 (FI-14) (10 µM), or ML-9 (10 µM) for an additional 30 minutes. Stiffness measurements at optimal length *L*_O_ were collected continuously during ten minutes of stimulation with the alpha-agonist phenylephrine (PE, 10 µM) at a maximally effective concentration.

Tissue stiffness was measured by small-amplitude (1%), high-frequency (40 Hz) sinusoidal length perturbations as previously described [4], [29]. This regime has previously been shown to measure total tissue stiffness without breaking actin-myosin crossbridges [4]. With oscillatory stretching, tissue stiffness *E* = *E*’+*iE*”, where *E*’ is the elastic or storage modulus (the in-phase component of the stiffness), E” is the viscous or loss modulus (the out-of-phase component of the stiffness), and *i* is the unit imaginary number. As reported previously [4], with this protocol the force and length signals are in-phase, and therefore the out-of-phase viscous component of the stiffness is negligible. Thus, the tissue’s elastic modulus, or material stiffness, *E* is calculated as the ratio of the stress *σ* to the strain *ε*, *E* = (Δ*F*/*A*)/(Δ*L*/*L*_O_), where Δ*F* is the amplitude of the force response to the cyclic stretches, *A* is the cross-sectional area, and Δ*L* is the amplitude of the cyclic stretches. When stretched to optimal length, the walls of the ring collapse, allowing the cross-sectional area *A* to be calculated as twice the product of the wall thickness *h* and the axial length of the ring *l*, *A = 2hl*.

### Western Blot Analysis and Measurement of LC20 Phosphorylation

Quick-frozen tissues were homogenized and processed at 4°C as previously described [25]. The homogenization buffer was designed to preserve phosphoproteins, and it consists of 20 mM MOPS, 4% SDS, 10% glycerol, 10 mM DTT, 20 mM β-glycerophosphate, 5.5 µM leupeptin, 5.5 µM pepstatin, 20 KIU aprotinin, 2 mM Na_3_VO_4_, 1 mM NaF, 100 µM ZnCl_2_, 20 µM AEBSF, and 5 mM EGTA. Western blot densitometry was performed using Odyssey Infrared Imaging System (LI-COR Biosciences, Lincoln, NE). Glycerol-urea gel electrophoresis was performed to separate nonphosphorylated, monophosphorylated, and diphosphorylated light chains as previously described [22], and LC20 phosphorylation was calculated as a ratio of phosphorylated LC20 to total LC20. LC20 phosphorylation was examined 30s into stimulation with PE, before both stiffness and contractile force reach their maximal values, as we have shown previously that this time point corresponds to maximum LC20 phosphorylation in aortic smooth muscle [30].

### Reagents and Antibodies

Agonists used were LPA from Cayman Chemical (Ann Arbor, MI) and phenylephrine from Sigma-Aldrich. PP2 was purchased from EMD Biosciences (La Jolla, CA); FAK inhibitor 14 was purchased from Santa Cruz Biotechnology (Santa Cruz, CA); and ML-9 was purchased from Calbiochem (La Jolla, CA). General laboratory reagents were of analytical grade or better and purchased from Sigma-Aldrich and Bio-Rad Laboratories (Hercules, CA). The following primary antibodies were used: phospho-tyrosine (mouse, 1∶500) from BD Biosciences (San Jose, CA); phospho-FAK Y925 (rabbit, 1∶200), phospho-paxillin Y118 (rabbit, 1∶250), and phospho-CAS Y165 (rabbit, 1∶250) from Cell Signaling Technology (Danvers, MA); vinculin (mouse V4505, 1∶400) from Sigma-Aldrich; and a-tubulin (rabbit, 1∶50,000 from Abcam (Cambridge, MA). For immunofluorescence experiments, goat anti-rabbit and goat anti-mouse Alexa Fluor 488 and Alexa Fluor 568 (1∶1,000; Invitrogen) were used as secondary antibodies. For Western blot experiments, Goat Oregon Green 488 or Alexa Fluor 568 labeled anti-rabbit or anti-mouse IgGs were used as secondary antibodies (1∶1,000; LI-COR Biosciences, Lincoln, NE).

### Statistical Analysis

Data are reported as means +/− standard error. Differences between individual means were determined by two-tailed Student’s t-test. Differences were considered significant at p<0.05.

## Results

### VSMC Cortical Stiffness is Modulated by FAs and Contractile Activation

**Figure 1:**
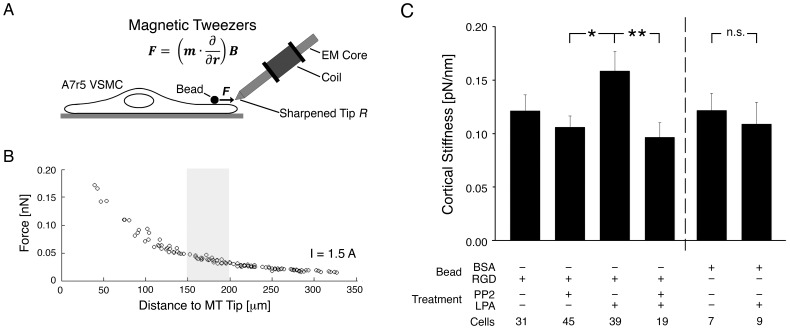
Cortical stiffness measurement in VSMCs. (A) Controlled force pulses generated by magnetic tweezers displace aortic VSMC-adherent, RGD-coated superparamagnetic beads (2.8 µm) to measure the stiffness of the bead-focal adhesion-cortical cytoskeleton linkage (see Methods). The force *F* exerted on a bead depends on the induced magnetic moment in the bead *m* and the spatial gradient of the magnetic field *B*, which depends critically on the sharpness of the probe’s tip, characterized by its radius of curvature *R*. (B) Calibration curve relating the force exerted on a bead (d = 2.8 µm) to its distance from the MT tip (R = 40 µm) and the current through the electromagnet solenoid (I = 1.5 A). The gray box denotes the operating range used for the MT experiments, i.e. the distance that is set between the MT tip and a bead before pulling commences. (C) Mean cortical stiffness increases with LPA stimulation in a PP2-attenuated manner. Right: BSA beads, which do not bind integrins but adhere nonspecifically, do not register an increase in stiffness with LPA. *p<0.05, **p<0.01, n.s. – not significant, unpaired, two-tailed Student’s t-test, assuming unequal variances.

To test the role of FAs in regulating cell stiffness, minimizing the contribution of the ECM, we measured cortical stiffness of serum-starved A7r5 VSMCs with MT (Figure 1A). For these experiments, the position of the magnetic probe tip was adjusted to be in the more linear range of the force-distance calibration curve (Figure 1B). The low forces used here (∼50 pN) confine stiffness measurements to the cortex of the cell [16]–[19], encompassing the bead-integrin attachment as well as the underlying FA and cortical cytoskeleton.

Mean cortical stiffness (Figure 1C) was measured with RGD-coated beads in order to bind integrins (0.122±0.015 pN/nm). A7r5 cells do not respond to classical vasoconstrictors, but stimulation with non-mitogenic LPA mimics vasoconstrictors such as PE since it increases myosin phosphorylation to activate myosin [22], [31], [32]. LPA increased stiffness (0.159±0.018 pN/nm) significantly (p<0.01), but pre-treatment before LPA stimulation with PP2, a specific small molecule inhibitor that prevents FA remodeling by inhibiting Src family kinases [22], [33]–[35], reduced cortical stiffness to untreated baseline levels. These results directly implicate the FA and the surrounding cortical cytoskeleton in agonist-induced increases in stiffness of the bead-ECM-cytoskeleton linkage in A7r5 VSMCs.

As a control, we performed experiments with bovine serum albumin (BSA)-coated beads, which adhere nonspecifically to cell membranes [36]. There was no difference between cortical stiffness measured with RGD beads and BSA beads in the absence of a stimulus; however, measurements of stiffness with the BSA beads did not exhibit the increase in stiffness observed with RGD beads following LPA stimulation of the A7r5 VSMCs (Figure 1C), thereby confirming the existence of a functional focal adhesion linkage between the RGD beads and the underlying cortical cytoskeleton.

### LPA Stimulation Increases FA Size in a PP2-attenuated Manner

**Figure 2:**
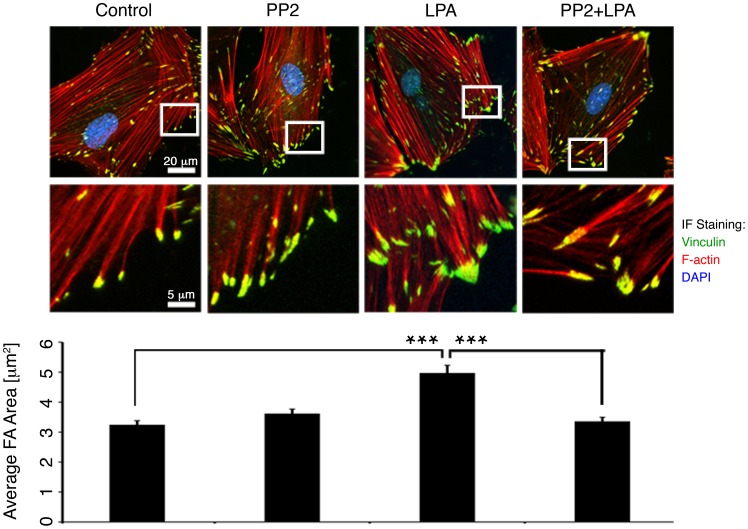
LPA stimulation increases FA size in a PP2-sensitive manner. Top: Deconvolution microscopy of FAs. Representative FAs are shown in an expanded view in the insets. All FAs in each cell, not just those featured in the insets, were analyzed to determine the mean FA area. Scale bars: top, 20 µm; bottom, 5 µm. Bottom: Mean FA area is significantly greater in cells stimulated with LPA (n = 82 cells). ***p<0.001, unpaired, two-tailed Student’s t-test.

A larger FA would have greater stiffness as measured by the MT. We hypothesized that changes in FA dimensions might underlie our cortical stiffness observations since FAs of migrating cells grow in a force-dependent manner [37], and Src, together with FAK, is known to affect FA recycling and growth in nonmuscle cells [34]. Thus, we quantitated FA sizes using deconvolution immunofluorescent imaging of vinculin, a marker of FAs (Figure 2).

Mean FA size did not change with PP2 treatment alone. FAs were significantly larger in cells stimulated with LPA (4.97±0.26 µm^2^ versus 3.24±0.14 µm^2^ in control cells, a 55% increase) (p<0.001). This is consistent with effects reported in migrating cells upon myosin activation and suggests that a tension-induced increase in FA size in the presence of LPA increases cortical stiffness. Additionally, the growth in FA size with LPA was prevented by PP2, which is also consistent with the known effect of PP2 to prevent FA remodeling and growth in other cell types [33], [34]. Vinculin has multiple actin binding sites and is known to crosslink actin and the FA [38]; hence, increased vinculin staining may reflect additional actin-FA interactions and concurrent stiffening and strengthening of the FA and the surrounding cortical cytoskeleton. There was no statistically significant difference between the number of focal adhesions per unit area (0.054±0.003 µm^−2^) in any of the conditions examined.

### Contractile Filament Activation is a Component of Agonist-induced Aortic Tissue Stiffness

**Figure 3:**
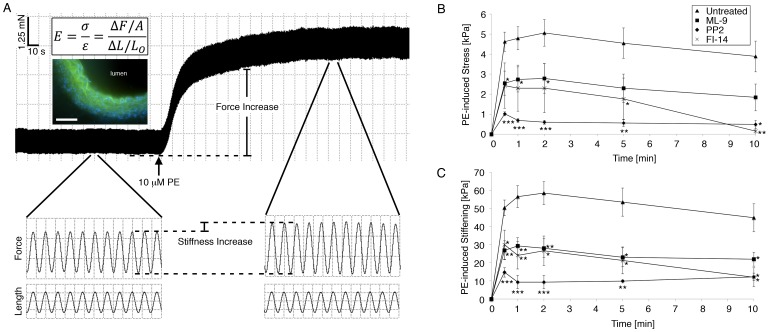
Aortic tissue stiffening during contractile stimulation is decreased by inhibition of Src, FAK, or MLCK. (A) Tissue stiffness *E* measured *in vitro* during PE-induced contraction at optimum length *L*_O_ with small-amplitude (1%), high frequency (40Hz) sinusoidal stretches Δ*L*. Box height and width for magnified traces: 0.5 mN, 5 µm, and 0.02 s. *Upper Inset*: Stiffness calculation. *Lower Inset*: Fluorescent micrograph of aortic ring in cross-section, used to determine ring thickness for calculation of cross-sectional area *A*. Green: autofluorescent elastic laminae. Blue: cell nuclei. Scale bar, 100 µm. (B) PE-induced stress is significantly lower when pre-treated with MLCK inhibitor ML-9, confirming the importance of myosin activation and contraction to vascular stiffness (n = 4). PE-induced stress is also significantly reduced when pre-treated with Src inhibitor PP2 and FAK inhibitor 14. (C) PE-induced stiffening is significantly reduced when pre-treated with MLCK inhibitor ML-9, confirming the importance of myosin activation to aortic stiffness. Stiffening is also significantly lower when pre-treated with Src inhibitor PP2 and FAK inhibitor 14, indicating a role for Src, FAK, and FA proteins in aortic stiffness. n = 10 untreated, 6 ML-9, 4 PP2, 5 FI-14 rings. *p<0.05,**p<0.01, ***p<0.001, unpaired, two-tailed Student’s t-test.

To extend our vascular smooth muscle cell studies to vascular tissue, we measured stiffness of mouse aorta rings *in vitro* with small-amplitude sinusoidal stretches that do not break crossbridges [4]. This stretching produces an in-phase force response that simplifies the stiffness calculation to a normalized ratio of the force response to the imposed length cycling (see Methods). The sample force trace (Figure 3A) illustrates the continuous measurement of both force generation (the rise of the trace) and stiffening (the widening of the force response with constant amplitude length perturbations) during stimulation with the vasoconstrictor PE. Additionally, the sample trace demonstrates the importance of smooth muscle cell activation as a modulator of aortic wall stiffness, as it represents roughly 20% of total stiffness. The remaining 80% of the stiffness of activated tissue equals the baseline, unstimulated stiffness (213+/−7 kPa), which is likely determined largely by the ECM. Changes in stiffness (“stiffening”) are plotted in Figure 3 as opposed to absolute stiffness, as the focus of this study is to examine the contribution of the smooth muscle cell component rather than the matrix component to stiffness.

Smooth muscle contractility is regulated by myosin light chain kinase (MLCK)-dependent myosin phosphorylation [39]. Rings pre-treated with the MLCK inhibitor ML-9 before PE stimulation exhibited a significant decrease in stress generation (43–53%) (p<0.05) (Figure 3B) and a significant decrease in stiffening (47–57%) (p<0.01–0.05) (Figure 3C). These results demonstrate that the actomyosin contractile apparatus of the VSMC is a major component of regulated blood vessel stiffness.

### Aortic Tissue Stiffening is Decreased by Inhibition of Src or FAK

To test the role of FA remodeling in regulating stiffness of the intact vessel wall, we measured the stiffness of aortic rings pre-treated with PP2 before PE activation. PP2 significantly decreased PE-induced stiffening (71–84%) (p<0.001–0.05) (Figure 3C).

These results are consistent with the concept that Src-dependent FA recycling is a significant regulator of aortic stiffness.

Unexpectedly, PP2 pre-treatment also significantly decreased PE-induced stress generation (78–88%) (p<0.001–0.05) (Figure 3B). At first glance, the magnitude of this decrease suggests that PP2 might directly alter smooth muscle contractility, possibly by an effect on Rho (via p190RhoGAP). RhoA/Rho-kinase signals downstream to MLC (myosin light chain) phosphatase, increasing MLC phosphorylation [40], [41]. We have previously published that the alpha-agonist PE increases myosin phosphorylation and activation [42] and that the concentration of PP2 employed here does not affect MLC phosphorylation in aortic tissue of the ferret [22]. We have now confirmed by urea gel analysis that PE induces MLC phosphorylation in mouse aorta, unaffected by PP2 (unstimulated, untreated: 0.218+/−0.046 mol PO_4_/mol LC; PE: 0.375+/−0.049 mol PO_4_/mol LC, p<0.05; PP2+PE: 0.362+/−0.039 mol PO_4_/mol LC, n.s., n = 5). Src is also known in some systems to regulate actin polymerization [40]. In vascular smooth muscle tissue, actin remodeling induced by PE has been shown to be largely cortical [6]. Thus, the inhibition of Src function by PP2 seems to involve solely the focal adhesion and the associated cortical cytoskeleton, presumably hindering the transmission of force from the contractile filaments across the focal adhesion.

We also measured stress and stiffness for aortic rings pre-treated with the specific small molecule inhibitor FAK inhibitor 14 before PE activation [43]. As with PP2, FI-14 also significantly decreased PE-induced stiffening (40–73%) (p<0.01–0.05) (Figure 3C) and stress generation (47–96%) (p<0.01–0.05) (Figure 3B). Taken together, these results implicate the FAK-Src signaling complex of the focal adhesion as a regulator of tension and stiffness in the aortic wall.

### Src- and FAK- Mediated Tyrosine Phosphorylation of FA Proteins Parallels Increases in Aortic Stiffness

**Figure 4:**
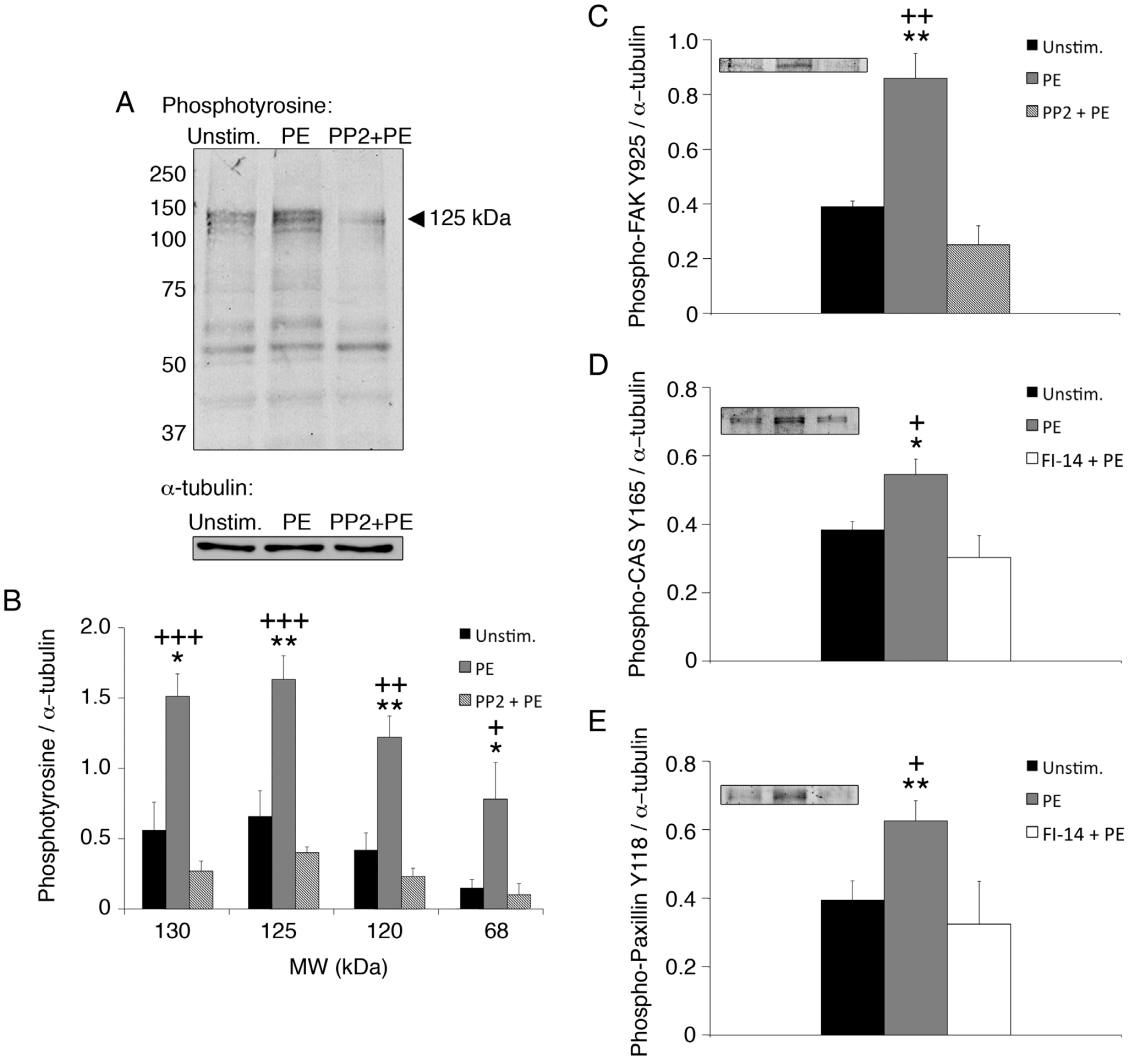
PE induces FAK and Src-mediated tyrosine phosphorylation of FA proteins in dVSMCs. (A) Typical blot, phosphotyrosine screening of mouse aorta tissue homogenates. PE increases tyrosine phosphorylation, and pre-treatment with Src inhibitor PP2 decreases tyrosine phosphorylation. (B) Mean densitometry of phosphotyrosine bands indicated. (C) Phospho-FAK Y925 increases in response to PE in a PP2-inhibitable manner, mean densitometry (n = 9 mice, 3 experiments). (D-E) Phospho-CAS Y165 and phospho-paxillin Y118 increase in response to PE in a FI-14-inhibitable manner, mean densitometry (n = 9 mice, 3 experiments). The brightness of the representative bands in Insets C-E has been uniformly altered for visual display; however, unaltered images were used for densitometry quantitation. *p<0.05, **p<0.01 vs. control, +p<0.05, ++p<0.01, +++p<0.001 vs. PP2+PE or FI-14+PE, unpaired, two-tailed Student’s t-test.

To confirm that PP2 inhibits Src-dependent FA signaling in aortic tissue, we performed phosphotyrosine screens of aorta homogenates. As seen in Figures 4A and 4B, PE increases tyrosine phosphorylation, and PE-induced increases in phosphotyrosine are inhibited by PP2, notably for bands at 130, 125, 120 and 68 kDa (p<0.001–0.05). These bands have been previously shown to correspond to focal adhesion proteins, including CAS, FAK, and paxillin, in aorta tissue from the ferret [22].

The p125 band co-stained with an antibody against 125-kDa FAK, a major FA signaling protein and Src binding protein [44]. A FAK Y925-site-specific phospho-antibody confirmed the presence of a significant PE-induced increase in phosphorylation and a significant inhibition of the PE effect by PP2 (p<0.01) (Figure 4C). FAK Y925 is a Src substrate that signals downstream to ERK [44], which is also known to be activated by PE in aortic tissue [22], [45], [46].

To further explore agonist-induced FA signaling, we examined phosphotyrosine levels in the presence of FAK inhibitor 14. Phospho-specific antibodies against p130 CAS Y165 and p68 paxillin Y118, both substrates of the FAK-Src signaling complex [44], demonstrated statistically significant PE-induced increases in phosphorylation that are significantly inhibited by FI-14 (p<0.01–0.05) (Figures 4D–E).

Thus, taken together, these results are consistent with the concept that, at the tissue level, vasoconstrictor activation initiates Src- and FAK-dependent FA signaling, which, in turn, regulates aortic stiffness and contractility.

## Discussion

The main finding of this study is that the FA, as a cell-matrix linker, via the FAK-Src signaling network, is a significant and regulated component of aortic stiffness and tone. As a first step towards determining whether the cytoskeleton-FA linkage modulates vascular tissue stiffness, we measured cortical stiffness on the surface of aortic smooth muscle cells. To the best of our knowledge, our use of MT to measure cortical stiffness in VSMCs is novel but complementary to other techniques employing larger forces to sample whole cell stiffness [36], [47]. Others have shown that the nanometer lengths and piconewton forces we used with the MT are sufficiently small and weak to restrict perturbations to several hundred nanometers into only the cortex of the cell [16]–[19]. The 2.8-µm beads used for these experiments, roughly the size of individual FAs in these cells, allow selective probing of individual FAs. Both our bead and tissue deflections reflect strains that are less than 1%, insufficient to disrupt crossbridges [4]. Although the MT force range can evoke local responses from individual FA proteins in vitro [48]–[51], these forces are insufficient to activate large-scale biological responses, such as micromyogenic responses in VSMCs [36], which could obscure cortical measurements. Pulling with the MT is in the lateral direction, in contrast to the perpendicular indentation or retraction of atomic force microscopy or the torsion of magnetic twisting cytometry. Lateral pulls are closer to the direction of insertion of actin stress fibers into the cortex [52] and, therefore, sample cortical stiffness in a physiologically relevant direction.

The MT experiments revealed that cortical stiffness is increased by contractile stimulation with LPA in a Src-dependent manner. Cell immunofluorescence imaging was employed to explain the effects of LPA and PP2 on cortical stiffness. The imaging data demonstrate that contractile activation with LPA induces FA growth, similar to what has been observed in migrating and spreading cells [31], likely due to increased intracellular load on FAs [37]. The A7r5s cells are strongly adherent to the coverslips and do not shorten when stimulated, although they do activate contractile signaling pathways as shown by myosin light chain phosphorylation [53]. Therefore, the increase in FA area is not due to a decrease in cell size, which might bring distant FAs into close proximity or merge them into a single unit. Additionally, our measurements in the tissue were made at constant length, which rules out such effects at that length scale. Other investigators have reported that the relationship between FA size (based on vinculin staining) and traction forces in non-migrating cells is linear, i.e., larger FAs correlate with larger traction forces [37], [54]. Since in our system of non-migrating smooth muscle cells we examined FA size using vinculin as a marker, larger FAs may also reflect increased vinculin cross-linking of actin to the FA [38]. Thus, one may speculate that larger FAs, and the additional actin associated with them, form a stiffer, stronger link between the inside and outside of the cell, which is necessary to stably transmit greater forces to a cell’s substrate [31]. Additionally, we have previously shown by differential centrifugation in aorta of the ferret that CAS and Src redistribute in response to agonist stimulation; therefore, similar remodeling of the FA likely accompanies FA growth in response to agonist in rodent aorta [22].

After demonstrating that FAs and especially the FAK-Src signaling network are important to the cortical stiffening of a VSM cell model during contractile stimulation, we scaled up to *in vitro* stiffness measurements of rodent aorta. We found that smooth muscle contraction accounts for roughly 20% of stiffness, with the remaining 80% likely being due to extracellular matrix. Others have published that the ECM plays an important role in determining tissue stiffness and that changes in the ECM that occur with age or disease can affect tissue stiffness [3], but the workings of the smooth muscle component of stiffness are not as well understood. We found that FI-14 inhibited ∼60% of vascular stiffening and ∼50–95% of stress generation during stimulation, and PP2 inhibited ∼70% of vascular stiffening and ∼80% of stress generation during stimulation without affecting myosin light chain phosphorylation, which, together with our examination of phosphotyrosine signaling in the FAK-Src pathway in this tissue, is consistent with the concept that the FA, including cortical cytoskeletal connections, is a major regulator of stiffness. This finding is noteworthy given that until recently FAs in contractile vascular smooth muscle embedded in blood vessel walls were considered to be relatively static structures, in contrast to their highly dynamic analogues in migrating cell types [37]. Similarly, tyrosine phosphorylation of FA proteins, particularly by FAK and Src, is the hallmark of their turnover in migrating, cultured cells [34], [37] but has only recently been studied in contractile smooth muscle [22], [55], [56]. Since essentially all other known signaling events regulating contractile filament activation in contractile VSMCs involve serine or threonine phosphorylations [39], tyrosine phosphorylation events in these cells appear to be largely linked to the dynamics of FA proteins and the associated cortical actin cytoskeleton.

**Figure 5:**
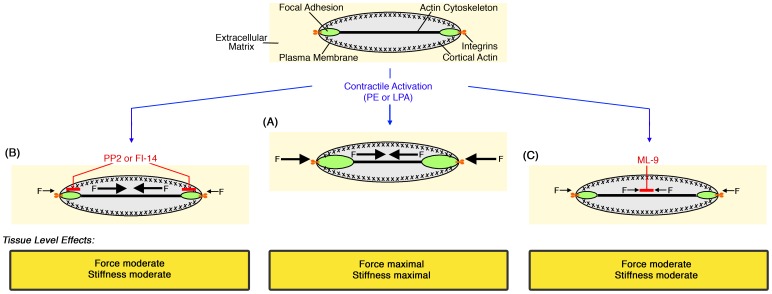
Model. (A) Tension-induced, Src- and FAK-mediated growth and remodeling of dynamic focal adhesions in aortic VSMCs leads to cell-matrix adhesion strengthening (cortical stiffening) in response to contractile stimulus. This strengthening is required for adequate force and stiffness transmission from the VSMC to the blood vessel wall. (B) Inhibition of Src with PP2 or FAK with FI-14 inhibits FA dynamics and growth, preventing reinforcement of the cell-matrix linkage. As a result, forces and stiffness generated by the activated VSMC cannot propagate efficiently to the tissue. (C) Inhibition of MLCK with ML-9 reduces contractile force and, as a result, lessens reinforcement of the cell-matrix linkage. Consequently, force and stiffness development in the aortic wall are reduced.

Taking these findings together, it appears that agonist-induced FA growth and remodeling entails strengthening of cytoskeleton-matrix linkages in VSM, permitting adequate force transmission from contracting cells to the vascular wall (see Model, Figure 5A). Prevention of FA/cortical cytoskeletal recycling via FAK-Src signaling, and by preventing FA growth, inhibits both force transmission and stiffness development (Figure 5B). Conversely, prevention of myosin activation directly inhibits force transmission and, by decreasing cytoskeletal tension, appears to also prevent FA growth, which decreases stiffness and further inhibits force transmission (Figure 5C). The fact that cortical actin and FA remodeling are concurrent in this system [6] might lead to changes in output via a FA-actin cytoskeleton “clutch” mechanism described for other cell types [57] that may control the efficiency of force transfer to the blood vessel wall.

In summary, our results point to the FA of the VSMC, specifically through interactions of FAK and Src, as a regulator of aortic vessel stiffness. We report that the FAs of these nonmigrating cells embedded in the wall of the aorta are regulated by vasoconstrictors and coordinate changes in force and stiffness development in the tissue. Given that increases in aortic stiffness are linked to cardiovascular disease, these results also point to the FA as a potential novel therapeutic target.
